# Th1 cell immune response in *Talaromyces marneffei* infection with anti-interferon-γ autoantibody syndrome

**DOI:** 10.1128/spectrum.03646-23

**Published:** 2024-03-18

**Authors:** Ye Qiu, Zheng-Tu Li, Wen Zeng, Jing-Lu Yang, Meng-Xin Tang, Yan Wang, Hao-Ru Wang, Yuanxiang Li, Yang-Qing Zhan, Shao-Qiang Li, Jian-Quan Zhang, Feng Ye

**Affiliations:** 1 Department of Respiratory and Critical Medicine, The Cancer Affiliated Hospital of Guangxi Medical University, Nanning, Guangxi, China; 2 State Key Laboratory of Respiratory Disease, National Clinical Research Center for Respiratory Disease, Guangzhou Institute of Respiratory Health, The First Affiliated Hospital of Guangzhou Medical University, Guangzhou, China; 3 Department of Respiratory and Critical Medicine, The First Affiliated Hospital of Guangxi Medical University, Nanning, Guangxi, China; 4 Department of Respiratory and Critical Medicine, The Eighth Affiliated Hospital, Sun Yat-Sen University, Shenzhen, Guangdong, China; Geisel School of Medicine at Dartmouth, Lebanon, New Hampshire, USA

**Keywords:** anti-interferon-γ autoantibody syndrome, *Talaromyces marneffei*, Th1 cell immune response, immunodeficiency, HIV-negative

## Abstract

Anti-interferon-γ autoantibody (AIGA) syndrome may be the basis of disseminated *Talaromyces marneffei* infection in human immunodeficiency virus (HIV)-negative adults. However, the pathogenesis of Th1 cell immunity in *T. marneffei* infection with AIGA syndrome is unknown. A multicenter study of HIV-negative individuals with *T. marneffei* infection was conducted between September 2018 and September 2020 in Guangdong and Guangxi, China. Patients were divided into AIGA-positive (AP) and AIGA-negative (AN) groups according to the AIGA titer and neutralizing activity. The relationship between AIGA syndrome and Th1 immune deficiency was investigated by using AP patient serum and purification of AIGA. Fifty-five HIV-negative adults with disseminated *T. marneffei* infection who were otherwise healthy were included. The prevalence of AIGA positivity was 83.6%. Based on their AIGA status, 46 and 9 patients were assigned to the AP and AN groups, respectively. The levels of Th1 cells, IFN-γ, and T-bet were higher in *T. marneffei*-infected patients than in healthy controls. However, the levels of CD4^+^ T-cell STAT-1 phosphorylation (pSTAT1) and Th1 cells were lower in the AP group than in the AN group. Both the serum of patients with AIGA syndrome and the AIGA purified from the serum of patients with AIGA syndrome could reduce CD4^+^ T-cell pSTAT1, Th1 cell differentiation and T-bet mRNA, and protein expression. The Th1 cell immune response plays a pivotal role in defense against *T. marneffei* infection in HIV-negative patients. Inhibition of the Th1 cell immune response may be an important pathological effect of AIGA syndrome.

**IMPORTANCE:**

The pathogenesis of Th1 cell immunity in *Talaromyces marneffei* infection with anti-interferon-γ autoantibody (AIGA) syndrome is unknown. This is an interesting study addressing an important knowledge gap regarding the pathogenesis of *T. marneffei* in non-HIV positive patients; in particular patients with AIGA. The finding of the Th1 cell immune response plays a pivotal role in defense against *T. marneffei* infection in HIV-negative patients, and inhibition of the Th1 cell immune response may be an important pathological effect of AIGA syndrome, which presented in this research could help bridge the current knowledge gap.

## INTRODUCTION

*Talaromyces marneffei* (*T. marneffei*) infection is regarded as a common and indicative opportunistic infection associated with immunosuppression in Southeast Asia, particularly in acquired immunodeficiency syndrome (AIDS) patients with CD4^+^ T-cell counts less than 200 cells/mL (1, 2). Due to the high mortality of talaromycosis and its rapidly increasing morbidity in the last 10 years, a global call has been sent out for clinical progress in its treatment, and the challenge of *T. marneffei* is being addressed by researchers worldwide (3–5). However, the pathogenesis of *T. marneffei* infections among HIV-negative individuals is unclear. Past studies have shown that low levels of CD4^+^ T and natural killer cells and a change in the immunological balance of Th17 and Treg cells may be involved in the pathogenesis of *T. marneffei* infections among HIV-negative individuals (1, 6). Surprisingly, we found that anti-interferon-γ autoantibody (AIGA) syndrome (also named AIGA disease and adult-onset immunodeficiency) was the most common underlying condition in HIV-negative *T. marneffei* infection patients and was an independent risk factor for severe, disseminated, refractory, persistent, and relapsed *T. marneffei* infections in our previous studies (4, 7–11). This suggests that AIGA syndrome may play a critical role in the pathogenesis of and be a predisposing factor for *T. marneffei* infections. However, current studies merely suggest that potential immune deficiency mechanisms in AIGA might be related to the fact that AIGA syndrome patient serum can inhibit STAT1 phosphorylation and IL-12 production in peripheral blood mononuclear cells (PBMCs) (12) and damage the antimicrobial function of macrophages (13) by impeding the IL-12/IFN-γ axis, causing microbial clearance failure.

The Th1 cell immune response has been demonstrated to play a pivotal role in defense against other fungal pathogens, including *Cryptococcus neoformans*, *Pneumocystis pneumonia, Candida albicans,* and *Aspergillus fumigatus* (14, 15). However, the Th1 cell immune response in *T. marneffei* infection, the effect of AIGA on the Th1 cell immune response, and the mechanism underlying their involvement in immune regulation are not clearly understood. Thus, this study was conducted by purifying AIGA from the serum of patients with AIGA syndrome to clarify the role of the Th1 cell immune response in HIV-negative patients with *T. marneffei* infection and to determine the effects of AIGA on the Th1 cell immune response.

## MATERIALS AND METHODS

### Study design

This was a multicenter prospective study (Chinese Clinical Trial Registry, ChiCTR2000029306) of HIV-negative individuals with *T. marneffei* infection recruited between September 2018 and September 2020 from four academic centers in Guangdong and Guangxi, China. Informed consent was obtained from all participants and their immediate family members. The Institutional Ethics Review Board of the First Affiliated Hospital of Guangzhou Medical University approved the study (reference number 2019026), and the First Affiliated Hospital of Guangxi Medical University approved the study (2020.KY-E-044), which was conducted by the guidelines of Good Clinical Practice and the Declaration of Helsinki.

The inclusion criteria for patients were as follows: (i) aged ≥18 years; (ii) laboratory evidence of HIV-negative status; (iii) except for AIGA syndrome, no other obvious immunosuppressive conditions, such as genetic defects, autoimmune diseases, glucocorticoid and/or immunosuppressive therapy, malignant tumors, diabetes or organ transplantation; (iv) presentation with histologically proven and culture-proven *T. marneffei* infections; and (v) provided informed consent for participation.

**Fig 1:**
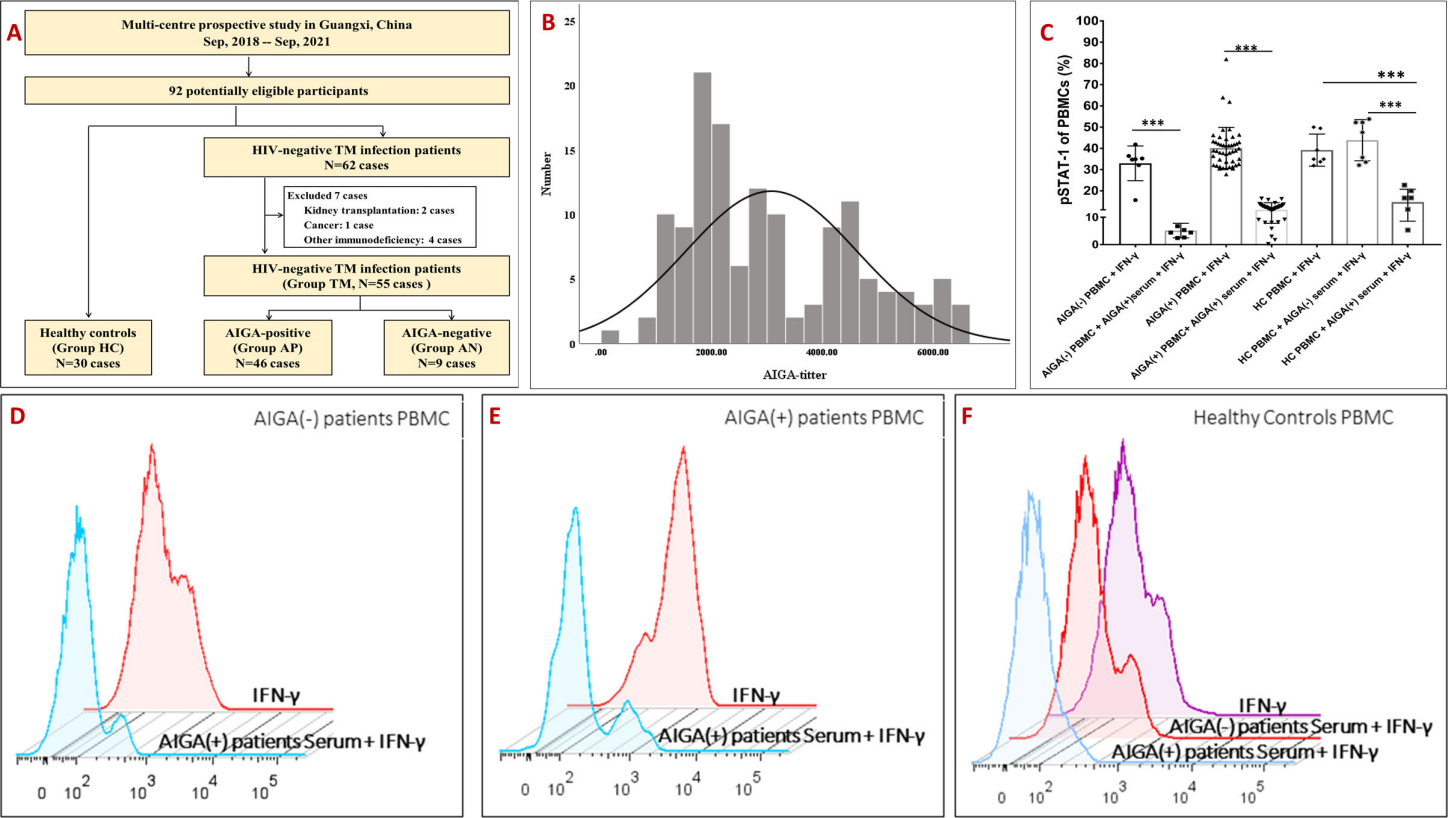
(**A**) Flow diagram of the recruitment of patients with *T. marneffei* infection. (**B–F**) The titer and neutralizing activity of AIGA. AIGA-positive serum from *T. marneffei*-infected patients showed good neutralizing activity *in vitro*. (**B**) Based on the 99th percentile of the AIGA titers in 103 healthy volunteers, the cutoff value for AIGA positivity was set at 6,520.57 ng/mL. (**C**) The neutralizing activity of AIGA was assessed by observing the impact of patient serum on IFN-γ-induced STAT-1 phosphorylation (pSTAT1) in PBMCs isolated from AIGA-positive patients, AIGA-negative patients, and healthy control volunteers by flow cytometry. (**D**) AIGA-positive serum inhibited IFN-γ-induced STAT-1 phosphorylation in PBMCs obtained from AIGA-negative *T. marneffei*-infected patients. (**E**) AIGA-positive serum inhibited IFN-γ-induced STAT-1 phosphorylation in AIGA-positive *T. marneffei*-infected patient PBMCs. (**F**) AIGA-positive serum inhibited IFN-γ-induced STAT-1 phosphorylation in PBMCs from healthy controls. However, serum from AIGA-negative patients did not inhibit IFN-γ-induced STAT-1 phosphorylation in PBMCs from healthy controls. In addition, when the AIGA-positive patient PBMCs were washed free of patient serum, they exhibited normal IFN-γ-induced STAT-1 phosphorylation. The volume of serum used for the intervention was 200 µL/mL. The data are expressed as the median. The comparisons were made by the Kruskal‒Wallis H test [one-way analysis of variance (ANOVA) on ranks]. *P < 0.05, **P < 0.01, ***P < 0.001. Group TM, *T. marneffei* infection group; Group AP, AIGA-positive individuals with *T. marneffei* infection; Group AN, AIGA-negative individuals with *T. marneffei* infection; HC, healthy control group; AIGA, anti-IFN-γ autoantibody; pSTAT1, phosphorylated STAT1.

Whole blood samples were obtained from all the participants. Samples from patients with *T. marneffei* infection were collected at the time the infection was diagnosed and before the start of antifungal treatment and AIGA immunotherapy. The titer and neutralizing activity of AIGA were detected in all participants. Based on the titer and neutralizing activity of AIGA, we divided the *T. marneffei*-infected patients into an AIGA-positive group (AP group) and an AIGA-negative group (AN group). A total of 103 healthy volunteers were recruited from the physical examination centers of the same hospitals to define the cutoff value for AIGA positivity. Thirty healthy volunteers were randomly selected as the healthy control group (HC group). The enrollment flow chart is shown in Fig. 1A.

We followed the patients after the first diagnosis and/or during the active stage of *T. marneffei* infection. All patients were followed up until 30 May 2021, or until death. Complete medical records, physical examinations, routine clinical laboratory tests, and AIGA titers were obtained for all patients.

### Clinical outcomes definitions

The clinical course of *T. marneffei* infection was divided into the following five categories: (i) curing (complete improvement of clinical symptoms after antifungal treatment and no recurrence during the 6-month follow-up); (ii) remission (partial improvement of clinical symptoms after antifungal treatment); (iii) persistent infection (deterioration or no improvement of clinical symptoms after treatment); (iv) recurrent infection (improvement of clinical symptoms, no pathogen detected after treatment, followed by the reappearance of *T. marneffei* infection signs, and/or a positive pathogen test result); and (v) death.

### Preparation of serum and PBMCs

Whole blood samples from participants were collected in ethylenediaminetetraacetic acid-treated tubes and inert separation gel vacuum procoagulant collective tubes. Serum was obtained by centrifugation of procoagulant blood and frozen at −80°C. PBMCs were separated by Lymphoprep (Stemcell Technologies, Canada) centrifugation and frozen at −196°C in liquid nitrogen. Briefly, fresh blood samples were mixed with an identical volume of phosphate-buffered saline (PBS) and carefully placed on the surface of the Lymphoprep separation medium. After centrifugation at 500 × *g* for 20 min at 28°C, the PBMCs were collected at the interphase and washed with PBS by centrifugation for 10 min at 300 × *g*.

### AIGA assay and assessment of the neutralizing activity of AIGA

The AIGA in the serum was measured by an enzyme-linked immunosorbent assay (ELISA) kit (Cloud-Clone Corp., Wuhan, China). Throughout the process, we strictly adhered to the manufacturer’s protocol regarding the preparation and testing of the standards and samples (9). The test method details for AIGA are provided in the Supplemental Materials and Methods. The neutralizing activity of AIGA was measured by flow cytometry analysis of IFN-γ-induced STAT-1 phosphorylation (pSTAT-1) in PBMCs (12).

### Positivity cutoff values of AIGA

The normal range for the AIGA titer was estimated using the log-normal distribution and was defined by the 99th percentile from 103 healthy volunteers (10). The mean titer of AIGA among these 103 healthy volunteers was 3,033.81 ± 1,576.31 ng/mL, and the 99th percentile concentration was 6,520.57 ng/mL. Therefore, the positivity cutoff value for AIGA was defined as an AIGA titer exceeding 6,520.57 ng/mL.

### Diagnostic criteria for AIGA syndrome

To diagnose AIGA syndrome, the following two conditions must be met simultaneously: (i) the detection titer of AIGA was outlying the 99th percentile from the 103 healthy volunteers (6,520.57 ng/mL) (10) and (ii) the neutralizing activity of AIGA indicated that it has neutralizing activity, which showed that it can inhibit IFN-γ-induced pSTAT-1 (12).

### Purification of AIGA from AIGA syndrome patient serum

The purification of AIGA from AIGA syndrome patient serum was performed as described in a previous study (16). Recombinant human IFN-γ (0.5 mg, Cloud Clone) was incubated with HiTrap NHS-Activated HP affinity columns (Cytiva) according to the manufacturer’s instructions. The unbound IFN-γ was removed by washing, and the columns were equilibrated with neutral buffer at a rate of 3 drops/s. Serum from either patient or control serum was diluted 1:2 with neutral buffer and then incubated with columns for 30 min. Uncoupled components were eluted in elution buffer (0.2 M NaHCO3, 0.5 M NaCl, pH 8.3) with 2–3 column volumes of elution buffer (100 mM glycine, 0.5 M NaCl, pH 3.0), and then we replaced the solution with PBS in Ultra15 (Millipore). Then, we measured the titer of AIGA with an ELISA kit (Cloud-Clone Corp.). The neutralizing activity of AIGA was measured by flow cytometry analysis of IFN-γ-induced pSTAT-1.

### AIGA-positive patient serum or purified-positive AIGA intervention

Purified positive AIGA was obtained from the serum of AIGA syndrome patients. AIGA-positive serum samples were serially diluted (1:5; 1:50; and 1:500). PBMCs from participants, including HIV-negative *T. marneffei*-infected patients and healthy volunteers, and H9 human T lymphocytes (BNCC, BNCC339983) were prepared at 1 × 10^7^ cells/mL and cultured in complete Roswell Park Memorial Institute-1640 medium containing 10% fetal bovine serum (Gibco) and 1% penicillin/streptomycin (Gibco) with or without purified positive AIGA or serially diluted AIGA-positive patient serum for 24 h. The cells were cultured in a humid atmosphere (5% CO_2_, 37°C).

### Cytokine, quantitative real-time PCR, Western blot analysis, and flow cytometry

The details of the cytokine analysis, quantitative real-time PCR, Western blot analysis, and flow cytometry are provided in the Supplemental Materials and Methods.

### Statistical analysis

The data are expressed as the median ± interquartile range. The Mann‒Whitney test was used for comparisons between the two groups. Kruskal‒Wallis one-way analysis of variance (ANOVA) on ranks or the Wilcoxon rank sum test was used to compare three or more groups. Correlation analysis was performed using Spearman’s rank correlation coefficient. We used SPSS (version 25.0) and GraphPad Prism (version 7, La Jolla, CA, USA) for statistical analysis and to prepare the graphs. The results with a *P* value < 0.05 were considered significant.

## RESULTS

### Baseline demographics and characteristics

During the 3-year study period, 62 HIV-negative patients with *T. marneffei* infection were observed. After excluding seven patients with underlying diseases, including kidney transplantation (two patients), cancer (one patient), and other immunodeficiencies (four patients), 55 HIV-negative *T. marneffei*-infected patients (TM group) were included. The enrollment flow chart and grouping procedure are shown in Fig. 1A. Among these 55 patients, 46 patients’ AIGA titers exceeded the cutoff value (6,520.57 ng/mL). Moreover, serum from these 46 patients had neutralizing activity, which showed that they can inhibit IFN-γ-induced pSTAT-1 (Fig. 1B through F). Ultimately, these 46 patients comprised the AP group. The AIGA titers of the remaining nine T. *marneffei*-infected patients were lower than the cutoff value and lacked neutralizing capacity; these patients comprised the AN group. In addition, when the PBMCs of AIGA-positive patients were washed free of the patient’s serum, they exhibited normal IFN-γ-induced STAT-1 phosphorylation.

The age and sex differences between the TM and HC groups were not significantly different (Table 1). The AIGA titer in the TM group (median 41,703.14 ng/mL, range 9,077.71–66,243.48 ng/mL) was greater than that in the HC group (median 2,328.90 ng/mL, range 1,927.03–4,283.35 ng/mL) (*P* < 0.001).

**TABLE 1:** Baseline demographics and AIGA titers of the 85 participants^*a*^

Variable	TM group (*n* = 55)	HC group (*n* = 30)	*P* value^*b*^
Age (years)	58 (49, 63)	54 (33, 57)	0.180
Sex, female, n (%)	26 (47.27)	16 (53.3)	0.593
AIGA-positive individuals, n (%)	46 (83.6)	0 (0)	**<0.001**
AIGA titer (ng/mL)	41,703.14 (9,077.71, 66,243.48)	2,328.9 (1,927.03, 4,283.35)	**<0.001**

^*a*^ TM, *Talaromyces marneffei*; HC, healthy control; AIGA, anti-IFN-γ autoantibody.

^*b*^ The bold in the table represents a *P* value < 0.05 and considered significant.

No apparent differences were observed between the AP and AN groups when patients were stratified by age or sex (Table 2). Higher leucocyte counts, neutrophil counts, erythrocyte sedimentation rates (ESRs), and C-reactive protein (CRP) levels were observed among the patients in the AP group than among those in the AN group (*P* < 0.05). Higher levels of immunoglobulin (IgG) were also observed in the AP group than in the AN group. Notably, patients in the AP group were more likely to have more involved sites and disseminated infection than those in the AN group (*P* < 0.05). In addition, the prognosis and outcomes in the AP group were worse than those in the AN group, especially for persistent infection, relapsed infection, and death (*P* < 0.05) (Table 2).

**TABLE 2:** Baseline demographics and clinical characteristics of the 55 participants^*a*^

Variable	AP group (*n* = 46)	AN group (*n* = 9)	*P* value^*b*^
Age (years)	58 (49, 63)	62 (50, 67)	0.218
Sex, female, n (%)	24 (52.2)	2 (22.2)	0.099
AIGA titer (ng/mL)	48,937.2 (22,202.7, 71,653.6)	3,664.9 (1,970.1, 5,723.7)	**<0.001**
No. of involved sites (n)	4 (3,6)	1 (1, 2)	**0.008**
Disseminated infection, n (%)	32 (69.6)	2 (22.2)	**0.022**
WBC (× 10^9^ cells/L)	14.9 (12.5, 20.3)	4.8 (4.2, 15.1)	**0.019**
N (× 10^9^ cells/L)	11.3 (8.6, 17.2)	2.7 (2.4, 10.8)	**0.035**
L (× 10^9^ cells/L)	1.9 (1.5, 2.7)	1.3 (0.7, 1.9)	0.053
HGB (g/L)	97.8 (77.0, 114.6)	78 (62.6, 120.4)	0.658
ESR (mm/h)	127.7 (105.3, 179.3)	78.0 (16.0, 100.0)	**0.037**
CRP (mg/L)	104.1 (68.9, 154.1)	55.7 (29.1, 141.6)	**0.031**
CD4^+^ T-cell count (cells/µL)	466 (257, 796)	606 (392, 855)	0.635
CD8^+^ T-cell count (cells/µL)	470 (250, 736)	422 (346, 822)	0.894
CD3^+^ T-cell count (cells/µL)	1,004 (645, 1,364)	1,246 (897, 1,550)	0.326
IgG (g/L)	28.4 (21.3, 36.9)	16.3 (13.2, 17.2)	**<0.001**
IgA (g/L)	2.6 (1.5, 3.6)	2.4 (2.3, 2.5)	0.821
IgM (g/L)	1.2 (0.9, 1.7)	0.9 (0.8, 0.9)	0.211
Prognosis and outcome			**0.007**
Cure	2 (4.3%)	3 (33.3%)	
Remission	20 (43.5%)	6 (66.7%)	
Persistent infection	7 (15.2%)	0	
Relapsed infection	10 (21.7%)	0	
Death	7 (15.2%)	0	

^*a*^ The data are expressed as the median ± interquartile range. Fisher’s exact test and the Kruskal–Wallis H test were used to determine the statistical significance of differences among the groups. *P* < 0.05. The data were collected under sterile conditions before the patient received antimicrobial therapy and during the active stage of the infection. Group AP = AIGA-positive individuals with *T. marneffei* infection. Group AN = individuals with *T. marneffei* infection in the AIGA-negative group. AIGA = anti-IFN-γ autoantibody, Ig=immunoglobulin. *The number of infecting pathogens is expressed as the median (minimum, maximum).

^*b*^ The bold in Table was mean a *P* value < 0.05 and considered significant.

### Th1 cells play a pivotal role in defense against *T. marneffei* infection in HIV-negative patients

**Fig 2:**
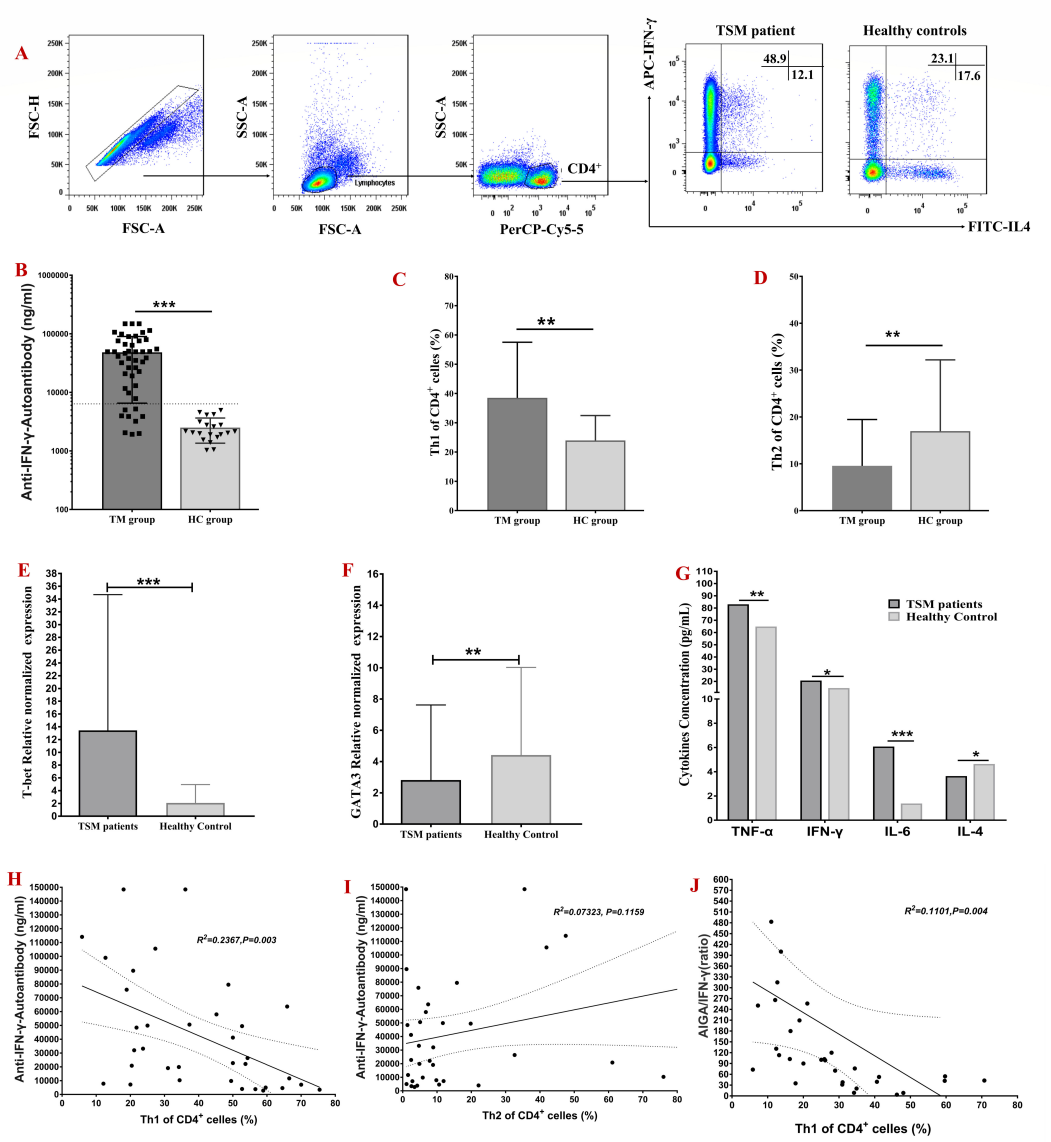
PBMCs were identified based on forward-scattered light (FSC) and side-scattered light (SSC) signals. Th1 cells were identified as CD4^+^IFN-γ^+^ cells, and Th2 cells were identified as CD4^+^IL-4^+^ cells. Representative scatter plots of the levels of Th1 cells and Th2 cells are shown (A). Th1 cell percentages, T-bet mRNA expression levels, and IL-6, IL-8, IFN-γ and TNF-α concentrations were considerably higher in the TM group than in the HC group (*P* < 0.05; C, E, G). However, the percentage of Th2 cells, the mRNA expression of GATA3, and the concentration of IL-4 in the TM group were lower than those in the HC group (*P* < 0.05; D, F, G). Finally, negative correlations were observed between the serum AIGA titer and the percentage of Th1 cells, the AIGA titer/INF-γ ratio and the percentage of Th1 cells in HIV-negative T. marneffei-infected patients (R^2^ = 0.2367, *P* = 0.003; R^2^ = 0.11, *P* = 0.004, respectively; H, I, J).

The percentages of Th1 and Th2 cells in 43 HIV-negative patients with *T. marneffei* infection and 20 healthy controls were examined. Th1 cell percentages, T-bet mRNA expression levels, and IL-6, IL-8, IFN-γ, and TNF-α concentrations were considerably higher in the TM group than in the HC group (*P* < 0.05; Fig. 2C, E, and G). However, the percentage of Th2 cells, the mRNA expression of GATA3, and the concentration of IL-4 in the TM group were lower than those in the HC group (*P* < 0.05; Fig. 2D, F, and G).

**Fig 3:**
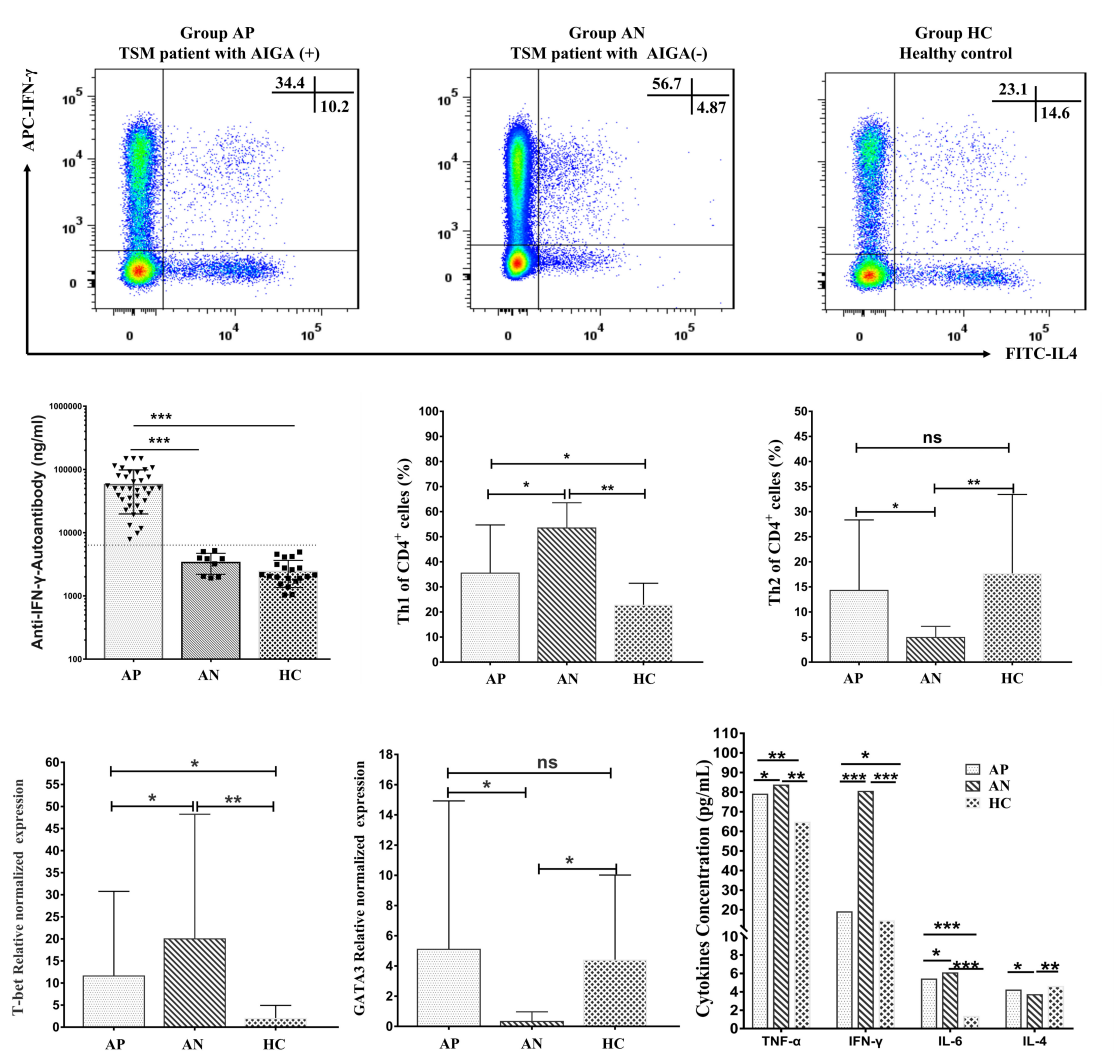
Comparison of the AIGA titers; the percentages of Th1 and Th2 cells among PBMCs; and the relative normalized expression of T-bet, GATA3, and cytokines among the AP, AN, and HC groups. The levels of circulating Th1 and Th2 cells were measured by flow cytometry. PBMCs were identified based on forward-scattered light (FSC) and side-scattered light (SSC) signals. Th1 cells were identified as CD4^+^IFN-γ^+^ cells, and Th2 cells were identified as CD4^+^IL-4^+^ cells. Representative scatter plots of the levels of Th1 cells and Th2 cells are shown. The relative normalized expression of T-bet and GATA3 was measured by quantitative real-time PCR. The concentrations of circulating IL-4, IFN-γ, TNF-α, and IL-6 were measured by ELISA. The correlations between the AIGA titer, the AIGA titer/INF-γ ratio, and the number of circulating Th1 cells in PBMCs were determined by Spearman’s rank correlation coefficient. Values represent the median with the interquartile range. Group TM = HIV-negative TM patients (*n* = 55); Group AP = HIV-negative, AIGA-positive TM patients (*n* = 46); Group AN = HIV-negative, AIGA-negative TM patients (*n* = 9); Group HC = healthy control volunteers (*n* = 30). The statistical analysis was performed using the Mann–Whitney U test. *P < 0.05, **P < 0.01, ***P < 0.001. TM, talaromycosis; Th, helper T cell; IL, interleukin; IFN, interferon; TNF, tumor necrosis factor; T-bet, T-box expressed in T cells; GATA-3, GATA binding protein 3.

Notably, the percentages of Th1 cells; the concentrations of IFN-γ, TNF-α, and IL-6; and the mRNA expression levels of T-bet were significantly lower in the AP group than in the AN group (*P* < 0.05). In contrast, the percentages of Th2 cells, the concentration of IL-4, and the mRNA expression levels of GATA3 were higher in the AP group than in the AN group (*P* < 0.05; Fig. 3).

Finally, negative correlations were observed between the serum AIGA titer and the percentage of Th1 cells, the AIGA titer/INF-γ ratio, and the percentage of Th1 cells in HIV-negative *T. marneffei*-infected patients (R^2^ = 0.2367, *P* = 0.003; R^2^ = 0.11, *P* = 0.004, respectively; Fig. 2H, I, and J).

Thus, the proliferation of Th1 cells in HIV-negative patients with *T. marneffei* infection was markedly increased, but this phenomenon may be inhibited when AIGA is present in patients with AIGA syndrome.

### AIGA can inhibit CD4^+^ T-cell pSTAT-1 and the Th1 cell immune response

Serum samples were isolated from 20 AIGA syndrome patients with *T. marneffei* infection. Then, we cultured the healthy control and AIGA syndrome patients’ PBMCs treated with or without AIGA syndrome patients’ serum for 24 h. Afterward, we collected the cells and stained them for CD4, STAT-1 phosphorylation (pSTAT1), IFN-γ, and IL-4 expression, which was detected by flow cytometry.

**Fig 4:**
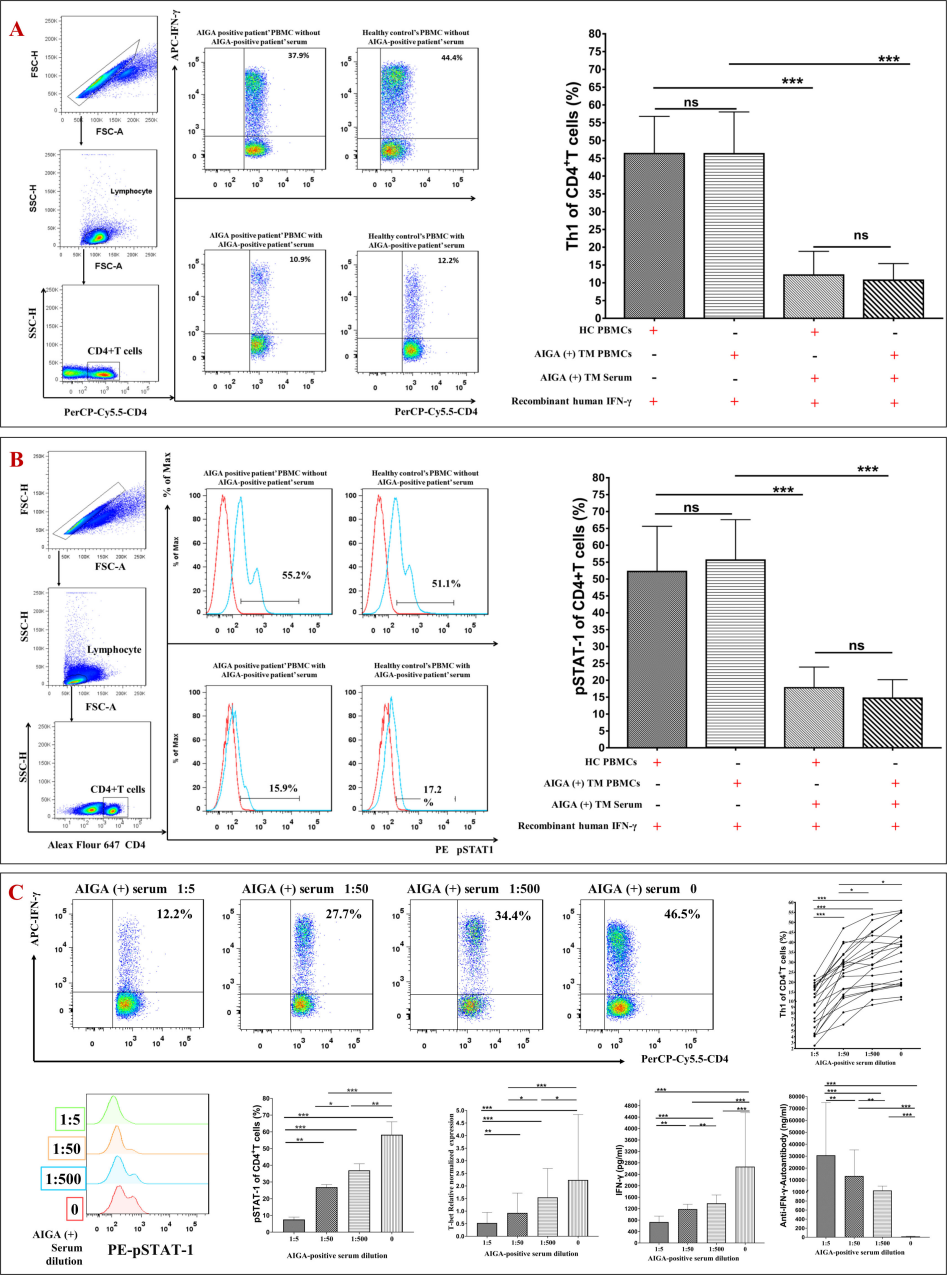
Serum from AIGA-positive patients inhibited STAT-1 phosphorylation in CD4^+^ T cells and the generation of Th1 cells *in vitro*. (**A**) and (**B**) We cultured PBMCs treated with or without AIGA-positive patient serum for 24 h. (**C**) We cultured healthy control PBMCs treated with serially diluted serum from 39 AIGA-positive patients (1:5, 1:50, and 1:500) or without serum from AIGA-positive patients for 24 h.

pSTAT1 expression in CD4^+^ T cells and Th1 cell differentiation were dramatically decreased not only in AIGA syndrome patient PBMCs but also in healthy control PBMCs after culture in AIGA syndrome patients’ serum (*P* < 0.001) (Fig. 4A and B). In addition, when PBMCs from AIGA syndrome patients were washed free of serum, they exhibited normal levels of CD4^+^ T-cell pSTAT1 and Th1 cell differentiation.

**Fig 5:**
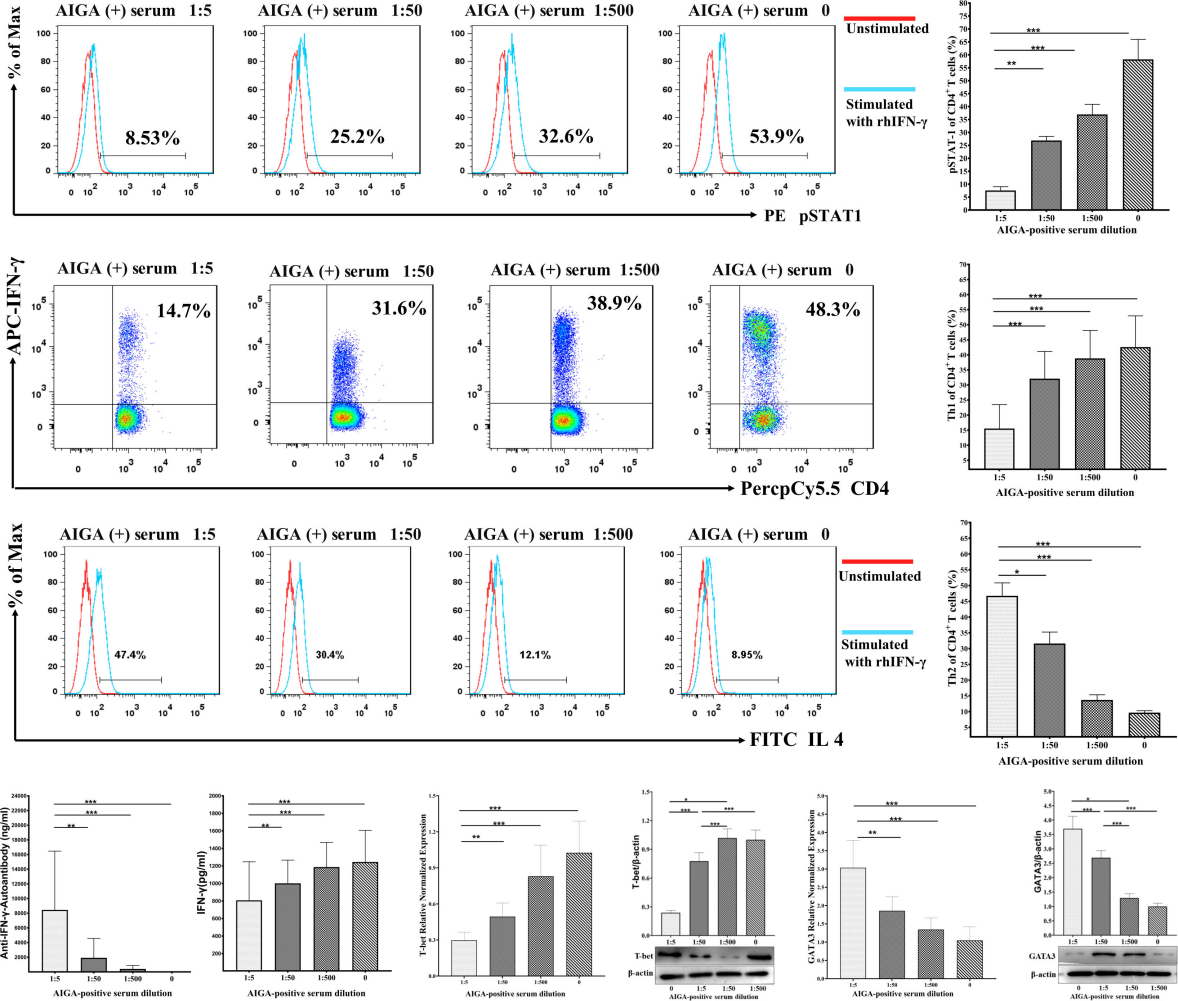
We cultured H9 human T lymphocyte lines with serially diluted serum from 39 AIGA-positive patients (1:5, 1:50, and 1:500) or without serum from AIGA-positive patients for 24 h. The cells were stimulated with 50 ng/mL PMA and 1 µg/mL ionomycin in the presence of GolgiStop (BD Pharmingen, San Diego, CA, USA) for 5 h. The cells were then collected and stained for CD4, pSTAT1, IFN-γ, and IL-4 expression, which was measured by flow cytometry. The relative (normalized) expression of T-bet was measured by quantitative real-time PCR. The concentrations of IFN-γ and anti-IFN-γ antibodies in the supernatant of cultured cells were measured by ELISA. Among the PBMCs, lymphocytes were identified based on FSC light and SSC light. Th1 cells were identified as CD4^+^IFN-γ^+^ cells. STAT-1 phosphorylation in CD4^+^ T cells was identified by CD4^+^pSTAT1^+^ staining. The data are expressed as the median. The statistical analysis was performed using the Mann–Whitney U test. *P < 0.05, **P < 0.01, ***P < 0.001; ns, not significant.

To assess the concentration-dependent inhibition of AIGA, we cultured PBMCs (Fig. 4C) and cultured H9 human T lymphocytes from 10 healthy controls (Fig. 5) treated with serially diluted serum from 20 AIGA-positive patients (0, 1:5, 1:50, and 1:500) for 24 h. Then, the cells were collected and stained for CD4, pSTAT1, IFN-γ, and IL-4 expression, which were detected by flow cytometry. Western blot analysis and quantitative real-time PCR were used to measure the protein and mRNA levels of T-bet and pSTAT1. Notably, we also found that the obvious inhibitory effect of AIGA syndrome patient serum on CD4^+^ T-cell pSTAT1 and the generation of Th1 cells was concentration dependent: the higher the concentration of AIGA was, the more pronounced the inhibition (*P* < 0.05).

### Purified AIGA can inhibit CD4^+^ T-cell pSTAT-1 and the Th1 Cell immune response *in vitro*

**Fig 6:**
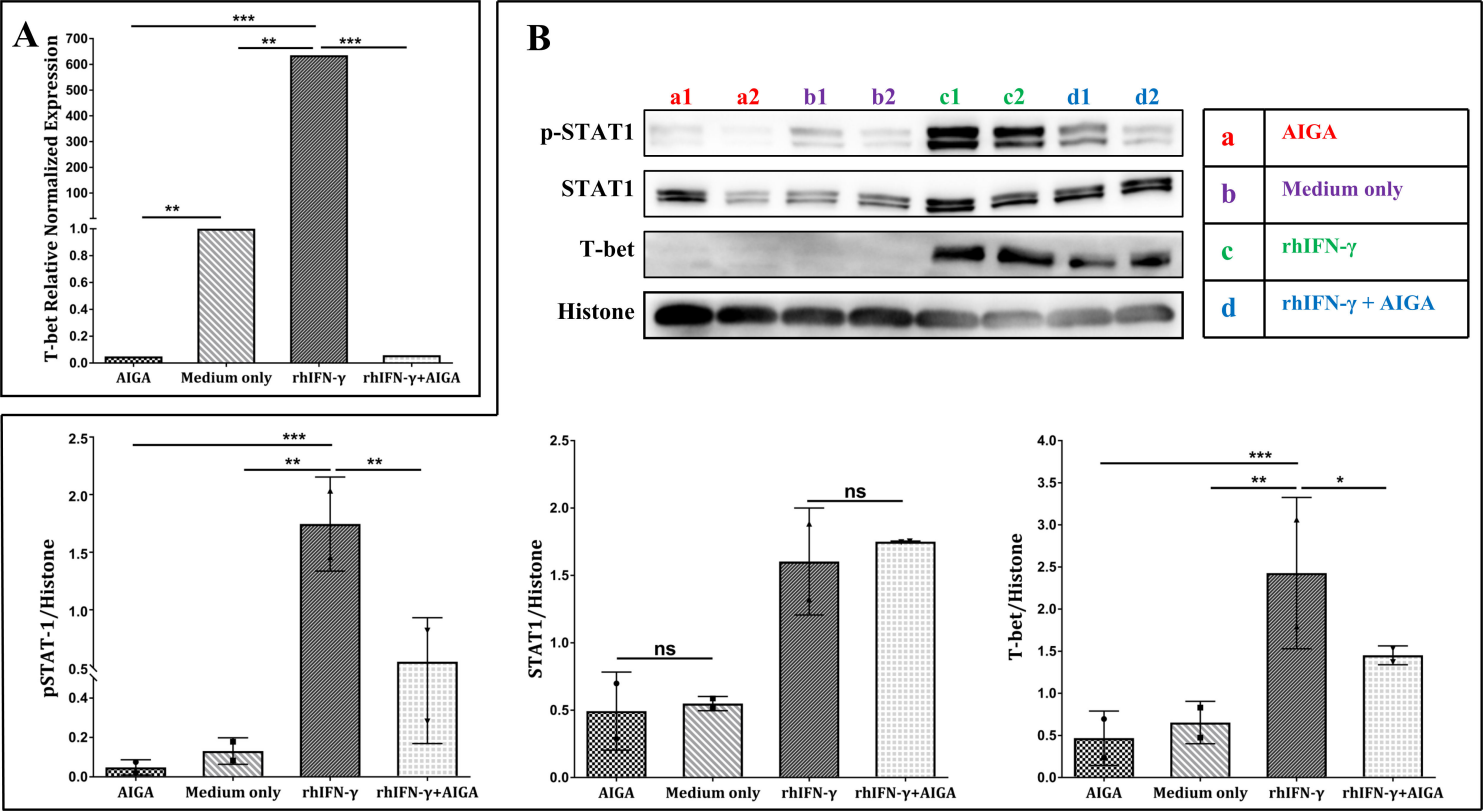
Purified AIGAs could directly contribute to inhibiting the generation of Th1 and CD4^+^ T-cell STAT-1 phosphorylation. We cultured H9 T cells pretreated with purified AIGAs or without treatment for 24 h. (**A**) Quantitative real-time PCR was used to measure the mRNA level of T-bet. (**B**) Western blot analysis was used to measure the phosphorylated T-bet and STAT-1 levels. In addition, recombinant human IFN-γ neutralized the inhibitory effect of purified AIGAs from AIGA-positive patients on CD4^+^ T-cell STAT-1 phosphorylation and the generation of Th1 cells. The data are expressed as the median. The statistical analysis was performed using the Mann–Whitney U test. *P < 0.05, **P < 0.01, ***P < 0.001; ns, not significant.

We cultured H9 T cells pretreated with or without purified AIGA for 24 h and found that purified AIGA inhibited CD4^+^ T-cell pSTAT1 and the mRNA and protein expression of T-bet, which is a critical nuclear transcription factor for Th1 cell generation. In addition, recombinant human IFN-γ neutralized the inhibitory effect of CD4^+^ T-cell pSTAT1 and the generation of Th1 cells from AIGA purified from AIGA syndrome patients (Fig. 6).

## DISCUSSION

In this research, we clarified the changes in Th1 cell immunity associated with *T. marneffei* infection, determined the effect of AIGA on Th1 cell immune responses, and investigated the effects of AIGA on Th1 cell immunity in adaptive immunity. In particular, we demonstrated the roles of the Th1 cell immune response and IFN-γ secretion in the defense against *T. marneffei* infection. We observed a remarkable increase in the number of Th1 cells and a decrease in the number of Th2 cells in peripheral blood when comparing HIV-negative *T. marneffei*-infected patients with healthy controls, which suggested that the Th1 cell immune response is important for *T. marneffei* infection in HIV-negative patients. Moreover, we found that the considerably high incidence rate (83.6%) of AIGA syndrome in adult patients with HIV-negative *T. marneffei* infections markedly affected the course of *T. marneffei* infection progression and patient prognosis, which was consistent with previous studies (4, 7, 9). Together, these results suggest that AIGA syndrome has a strong association with *T. marneffei* infection in HIV-negative adults and may be the immunodeficiency mechanism that enables this fungal infection, as well as other severe opportunistic infections, such as nontuberculous *Mycobacterium*, Varicella-Zoster virus, *T. marneffei*, and *Salmonella* spp. infections (7, 10).

AIGA syndrome was first identified as adult-onset immunodeficiency in disseminated and severe mycobacterial infections in 2012 (10) and was considered an autoimmune phenocopy of inborn genetic errors of the IL-12/IFN-γ axis in 2019 (11). To date, only a few studies on the immune deficiency mechanism of AIGA syndrome have focused on macrophages and PBMCs (13). Moreover, most of the current studies on immunodeficiency in AIGA syndrome patients simply use the serum/plasma of AIGA syndrome patients for intervention (10, 13, 17), but the serum/plasma components are complex, and it is not possible to directly confirm the direct effect of AIGA. Thus, it is necessary to purify the AIGA for further research. In the present study, for the first time, we explored the effects of AIGAs on the differentiation of Th1 and Th2 cells. We not only found that the serum and purified AIGA of HIV-negative *T. marneffei*-infected patients inhibited STAT1 phosphorylation and IL-12 production in PBMCs and CD4^+^ T cells, corroborating the results of previous studies but also observed the destruction of immune homeostasis between Th1 and Th2 activity and a remarkable inhibitory effect on Th1 cell differentiation, possibly mediated by the IFN-γ/STAT1/T-bet pathway, which damaged the antimicrobial function of effector T cells, causing a failure of microbial clearance. Thus, impaired Th1 cell immune response activity is an important component of the pathological effects of AIGA syndrome, and these findings provide further evidence of the pathophysiological processes that occur in patients with AIGA-associated immunodeficiency.

In this study, we found that AIGA syndrome patients had markedly greater inflammatory indicators (leucocyte counts, neutrophil counts, ESR, and CRP) and IgG levels than non-AIGA syndrome patients. In addition, the severity of *T. marneffei* infection, prognosis, and outcomes in AIGA syndrome patients were more severe and worse than those in AIGA syndrome patients, especially for double or multiple infections, disseminated infections, persistent infection, relapsed infection, and death, which is consistent with previous research (18). These issues may be related to immunodeficiency in AIGA syndrome patients; additionally, previous findings showed that macrophage dysfunction, the inhibition of STAT1 phosphorylation and IL-12 production, and Th1 cell immune injury, can cause microbial clearance failure. Thus, monitoring and predicting the titer and neutralizing activity of AIGA is crucial for assessing patient prognosis and host immunodeficiency severity.

The limitations of this study include the small proportion of patients in the AN group and the fact that the participants in this study were all from Guangxi and Guangdong. These results have successfully demonstrated the role of the Th1 cell immune response in the defense against *T. marneffei* infection and the effect of AIGA on Th1 differentiation and have implications for subsequent studies of the pathological effects of AIGA in AIGA syndrome.

In conclusion, the Th1 cell immune response is important for *T. marneffei* infection in HIV-negative patients. AIGA syndrome is the most crucial risk factor for severe *T. marneffei* infections in HIV-negative patients. The impaired activity of the Th1 cell immune response is an important component of the pathological effects that occur in AIGA syndrome, which is one of the most important pathogeneses of *T. marneffei* infections among HIV-negative individuals.

## AFTER EPUB

*[This article was published on 18 March 2024 with an error in the author affiliations. The affiliations were corrected in the current version, posted on 26 March 2024.]*

## SUPPLEMENTAL MATERIAL

Supplemental text spectrum.03646-23-s0001.docx Supplemental methods.

ASM does not own the copyrights to Supplemental Material that may be linked to, or accessed through, an article. The authors have granted ASM a non-exclusive, world-wide license to publish the Supplemental Material files. Please contact the corresponding author directly for reuse.
