# Supplementary material for: Th1 cell immune response in Talaromyces marneffei infection with anti-interferon-γ autoantibody syndrome
Source: Microbiol Spectr. 2024 Mar 18;12(5):e03646-23. doi: 10.1128/spectrum.03646-23 (PMC11064527; doi:10.1128/spectrum.03646-23)
Supplement: Supplemental text — Supplemental methods. [file spectrum.03646-23-s0001.docx]

**Online Supplement for Full Details on the Methods**

**Th1 cell immune response in *T. marneffei* infection with anti-interferon-γ autoantibody syndrome**

**Materials & Methods**

***Anti-IFN-γ Autoantibody (AIGA) Assay***

Serum and supernatant samples from each participant were obtained under sterile conditions before the patient received antifungal therapy for *T. marneffei* infection and during the active stage of the infection. Serum samples were retrieved from a serum bank and stored at −80 °C. AIGA levels were measured in all participants. All serum samples were tested at the first thaw. AIGA levels were measured with an enzyme-linked immunosorbent assay (ELISA) kit (Cloud-Clone Corp., Wuhan, China) as described in our previous research.

***IFN-γ, IL-4, IL-6, and TNF-α Assays***

All serum and supernatant samples were obtained under sterile conditions before the patients received antimicrobial therapy for *T. marneffei* infection and during the active stage of the infection. Serum samples were retrieved from a serum bank and stored at −80 °C. IFN-γ, IL-4, IL-6, and TNF-α levels were measured in all participants. All of the serum samples were tested at the first thaw. The measurement of IFN-γ, IL-4, IL-6, and TNF-α levels was performed using a human ELISA kit (Cloud-Clone Corp., Wuhan, China) according to the manufacturer’s instructions.

***Quantitative Real-Time Reverse Transcription PCR***

General RNA was acquired from PBMCs and stored at -80 °C with RNAiso Plus reagent according to the manufacturer’s protocol (9108, Takara). The quality and quantity of total RNA were evaluated using a spectrophotometer (NanoDrop 2000, Thermo Scientific). Complementary DNA (cDNA) was transcribed from total RNA using a PrimeScript RT reagent kit with gDNA Eraser according to the manufacturer’s protocol (RR047, Takara). Real-time PCR was conducted using SYBR® Premix Ex Taq™ II (RR820, TaKaRa). A typical 20 μL PCR mixture included 10 μL of SYBR® Premix Ex Taq™ II, 0.8 μL of each PCR primer, 2 μL of template cDNA, 0.4 μL of ROX Reference Dye, and 6 μL of ddH_2_O. The cycling conditions were 95 °C for 30 s, followed by 40 cycles at 95 °C for 5 s and 60 °C for 30 s. The reactions were performed on an ABI 7500 Real-Time PCR System. The primer sequences were as follows: β–Actin (forward 5ʹ-GTCATTCCAAATATGAGATGCGT-3ʹ, reverse 5ʹ-GTCATTCCAAATATGAGATGCGT-3ʹ) and T-bet (forward 5ʹ-AGGCTGAGTTTCGAGCAGTC-3ʹ, reverse 5ʹ-TGGCCTCGGTAGTAGGACAT-3ʹ). Relative gene expression was calculated using the 2^−ΔΔCt^ method.

***Western Blot Analysis***

After AIGAs were purified from PBMCs and H9 cells, the cells were collected and washed twice with PBS. The whole-cell protein content was extracted with ice-cold lysis buffer (50 mM Tris–HCl, pH 7.5, 150  mM NaCl, 2  mM ethylenediaminetetraacetic acid [EDTA], 1% (w/v) Nonidet P-40, 0.02% (w/v) sodium azide) supplemented with protease inhibitors (1  mM phenylmethylsulfonyl fluoride, PMSF). A Bradford assay was used to measure the protein concentrations. Equal amounts of protein were separated by 10% or 15% sodium dodecyl sulfate‒polyacrylamide gel electrophoresis. Then, the proteins were electrotransferred onto a nitrocellulose membrane (Millipore). Finally, the indicated primary antibodies were incubated with the membranes overnight at 4 °C.

The membranes were incubated with a primary antibody against T-Bet (Cell Signaling, Cat. No. 13232), GATA3 (Cell Signaling, Cat. No. 5852) and Histone (ABclonal, Cat. No. A2348), followed by incubation with the appropriate HRP-conjugated goat anti-rabbit IgG-HRP secondary antibody in iBind for 1 hour. Afterward, Tris-buffered saline containing 0.1% Tween 20 (TBST) was used to wash the membranes three times for 15 minutes per wash. Then, the membranes were incubated with ECL substrate solution (Beyotime Biotechnology, China) and visualized with autoradiography film. Protein loading was verified by the detection of histones using a histone H3 polyclonal antibody.

***Flow Cytometry***

*Evaluation of Th1 (IFN-γ^+^CD4^+^) and Th2 (IL-4^+^CD4^+^T) Cells Among PBMCs by Flow Cytometry*

Cells were stimulated with phorbol-12-myristate-13-acetate (PMA) (50 ng/mL) and ionomycin (2 μg/mL) under GolgiStop treatment at 37 °C in 5% CO_2_ for 5 h. After stimulation, cells were surface-stained with an anti-CD4 monoclonal antibody (Percp-cy5.5; BD Pharmingen) for 30 min at 4 °C and then fixed and permeabilized for 20 min at 4 °C. Then, the cells were incubated with anti-IFN-γ monoclonal antibodies (APC; BD Pharmingen) and anti-IL4 antibodies (FITC; BD Pharmingen) at 4 °C for 30 min. Th1 cells were defined as CD4^+^IFN-γ^+^ cells, and Th2 cells were defined as CD4^+^IL-4^+^ cells.

*Phospho-STAT-1 in PBMCs and H9 cells by Flow Cytometry*

To identify intracellular phospho-STAT-1 in PBMCs and H9 cells, an anti-CD4 monoclonal antibody (Alexa Fluor 647; BD Pharmingen) was used for 30 min at 4 °C. Intranuclear staining was performed with an anti–phospho-STAT1 (tyrosine 701) antibody (PE; BD Pharmingen). FACScan was used to collect data for flow cytometry (BD Biosciences), and FlowJo (Tree Star) was used for analysis.

The proportions of Th1 and Th2 cells as well as CD4^+^ T cells expressing phospho-STAT-1 among PBMCs obtained from 55 HIV-negative *T. marneffei*-infected patients and 20 HC subjects were analyzed by flow cytometry.
